# Tunable near-infrared epsilon-near-zero and plasmonic properties of Ag-ITO co-sputtered composite films

**DOI:** 10.1080/14686996.2018.1432230

**Published:** 2018-02-19

**Authors:** Chaonan Chen, Zhewei Wang, Ke Wu, Hui Ye

**Affiliations:** ^a^ State Key Laboratory of Modern Optical Instrumentation, College of Optical Science and Engineering, Zhejiang University, Hangzhou, P.R. China

**Keywords:** Indium tin oxide, co-sputtered composite films, tunable optical properties, plasmonics, 40 Optical, magnetic and electronic device materials, 103 Composites, 201 Electronics / Semiconductor / TCOs, 204 Optics / Optical applications, 302 Crystallization / Heat treatment / Crystal growth, 503 TEM, STEM, SEM

## Abstract

Series of co-sputtered silver-indium tin oxide (Ag-ITO) films are systematically fabricated. By tuning the atomic ratio of silver, composite films are manifested to have different microstructures with limited silver amount (<3 at.%). Two stages for film morphology changing are proposed to describe different status and growth mechanisms. The introduction of silver improves the preferred orientations of In_2_O_3_ component significantly. Remarkably, dielectric permittivity of Ag-ITO films is highly adjustable, allowing the cross-over wavelengths *λ*_*c*_ to be changed by more than 300 nm through rapid post-annealing, and thus resulting in tunable epsilon-near-zero and plasmonic properties in the near-infrared region. Lower imaginary permittivity compared with pure metal films, as well as larger tunability in *λ*_*c*_ than pure ITO films suggest the potentiality of Ag-ITO films as substituted near-infrared plasmonic materials. Extended Maxwell-Garnett model is applied for effective medium approximation and the red-shifting of epsilon-near-zero region with the increase of silver content is well-fitted. Angle-variable prism coupling is carried out to reveal the surface plasmon polariton features of our films at optical communication wavelength. Broad dips in reflectance curves around 52–56° correspond to the SPP in Ag-ITO films.

## Graphical abstract

**Figure:**
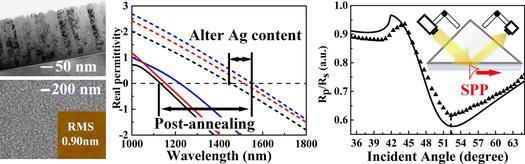

## 1. Introduction

Alternative plasmonic materials to conventional noble metals have been the forefront of intense study during the last decades, since feasible devices based on metallic building elements are impeded by their inherent bottlenecks, such as dissipative ohmic losses, non-tunable optical properties, and incompatibility with standard nanofabrication processes [1–3]. The extensive researches about alternatives mainly include highly-doped metal oxides, transition-metal nitrides, and two-dimensional materials [4–6]. Owing to their capability to support surface plasmons in the infra-red region, experimental realizations of promising plasmonic metamaterials and devices have been enabled [7–10]. One of the key issues for high-performing plasmonic materials is that their optical parameters can be manipulated at ease. In particular, the near-zero optical index (e.g. epsilon-near-zero, ENZ) is of paramount significance to not only the tunability of plasmonic resonances, but also some unique features allowing unprecedented optical phenomena [11,12], for instance, giant optical nonlinearity at the ENZ region [13,14]. The capability for plasmonic nanostructures to confine optical energy in sub-*λ* volumes also enables them to gain high energy concentration and enhance the optical nonlinearity. So, the ability of activating either localized plasmonic behaviors or propagating surface plasmon polaritons (SPP) modes makes plasmonic materials attractive in the field of bio- and chemical sensing, on-chip all-optical devices, information processing, nonlinear optics or other areas requiring respond speed and high efficiency [15].

Transparent conductive oxides (TCOs) can be recognized as metallic component and utilized as plasmonic materials in the near-infrared (NIR) region. Various TCO-based metamaterials and nanostructures have been proposed as substitutions to noble metals due to the natural advantages over metals in losses, easy manipulation by doping and fabrication [4,8,16–18]. Nevertheless, despite the attractiveness for the low-loss TCOs to operate in the NIR range, recent studies revealed that numerical assessments and analytical method seemed not to support their applicability. It was demonstrated that silver still outperforms most if not all NIR alternative plasmonic materials in terms of different figure-of-merit (FOM) [15,19]. In Ref. [15], FOM is defined as the ratio of plasma frequency and material loss, while in Ref. [19], it is expressed in more details as a two-dimensional graph between the degree of confinement and the propagation length since the trade-off of two factors needs to be considered. Although metal shows larger loss, it offers the best confinement-propagation balance compared with TCO even in the NIR region. Accordingly, the niche application of TCOs as NIR plasmonic materials requires the introduction of noble metals, especially the best candidate silver, to improve their overall performance.

However, previous research about metal-TCO-based materials focused on multilayer structures as well as their nanostructures [20–23]. Albeit the introduction of metal layer evidently improves the crystallinity, electrical properties and plasmonic strengths of the system, the drawbacks are obvious. When deriving optical refractive index of each layer, modeling of the multilayer systems requires intricate considerations of the interfaces between metal and TCO layers, which in present researches, neglect the unpredictable defects and mismatches of the boundaries, thus resulting in some deviations from the practical situations. Moreover, to evaluate the plasmonic properties of the multilayers, an effective index is needed by treating them as a single anisotropic layer [22,24]. Such approximation provides effective permittivity in parallel and perpendicular directions, in which one cannot obtain a single value for evaluation.

It is fascinating that if TCO and metals are equably blended in an isotropic composite layer, one can readily determine its single optical index by effective medium approximation. Reports about composite films and their potential applications have attracted attentions in the past few years, such as tunable plasmonic metamaterials, nonlinear devices and mostly, modulated electrode component [25–31]. Those theoretical and experimental results focused on visible [28,29], deep infrared [30], and terahertz [31] ranges. Consequently, here we propose an ITO-based binary composite films, by simply comprising small amount of Ag and substantial ITO. The promise of ITO-based films for visible transparent electrode has yet been fulfilled. However, in the NIR region where ITO acts as a favorable plasmonic material, no tunable composite materials are proposed for applications, especially for optical communications. As shown in Figure S1 (in the supplementary materials), the plasmonic performances of the proposed composite films in the NIR region are assessed using the combination of two-dimensional criteria mentioned above, which reveal their advantages in the realm of plasmon propagation and confinement over pure ITO to a certain extent. More importantly, the ENZ region of ITO is demonstrated to support large ultrafast third-order nonlinearity and an optically induced change in refractive index can also be acquired [14], which intrigues the interests in searching for the method of controlling the ENZ wavelength by the effect of metal component in composite films. In addition, a more updated theoretical analysis in structure and tunability in optical index for composite films are needed for better design and improvement of devices based on composite films. Hence, a comprehensive investigation of composite films operating around the telecommunication wavelength needs to be conducted, including structural analysis, growth mechanisms, optical analysis (permittivity, ENZ region, etc.), effective approximation for specific permittivity and ENZ-shifting simulation, as well as the verification of surface plasmons activated by 1550 nm light.

## 2. Experimental and theoretical basis

### 2.1. Fabrication

In our experiments, 200-nm-thick Ag-ITO composite films were fabricated by magnetron co-sputtering on glass and silicon substrates. The following characterizations are all performed on samples on silicon. Silver and ITO were sputtered simultaneously in the self-assembly chamber with Kurt J. Lesker Torus sputtering gun (Allegheny, PA, USA). The sputtering power was controlled to synthesize films with different silver atomic ratios. To control the deposition rate of each component, radio-frequency (RF) sputtering and direct-current (DC) sputtering were utilized for silver and ITO deposition, respectively. The power of DC sputtering was fixed at 300 W, while the RF power varied from 5 to 30 W. The substrate temperature was set at a relatively moderate value of 100 °C. Post rapid thermal annealing (RTA, Beijing Eaststar RTP-500, Beijing, China) in nitrogen atmosphere were performed to samples on silicon at 400 °C for 1 min.

### 2.2. Characterization

The surface and cross-sectional morphology were analyzed using field emission scanning electron microscopy (FESEM, Zeiss Ultra-55, Oberkochen, Germany) and transmission electron microscopy (TEM, Tecnai G2, Hillsboro, OR, USA ). The surface roughness was measured over 10 × 10 μm of sample surfaces by atomic force microscopy (AFM, Bruker Multimode, Karlsruhe, Germany) under ambient conditions. The atomic ratio and crystallinity of each element were retrieved from X-ray photoelectron spectroscopy (XPS, Krato Axis Supra, Manchester, UK) and X-ray diffractometer (XRD, Panalytical B V X-pert Powder, Westborough, MA, USA), respectively. The electrical properties were obtained from Hall measurements (Lakeshore 7604, Columbus, OH, USA). In addition, dielectric permittivities of the composite films were retrieved from spectroscopic ellipsometry (Semilab GES-5E, Tampa, FL, USA).

### 2.3. Drude–Lorentz model

The dielectric permittivity (real part *ɛ′* and imaginary part *ɛ*″) were extracted by fitting a Drude–Lorentz model to the measured ellipsometric data. This oscillation model well describes the optical phenomena emerging in these films. The following mathematical function (Equation (1)) describes the complex dielectric permittivity [32–35]:

(1) $$\varepsilon =\varepsilon^{\prime }+\text{i}\varepsilon^{\prime \prime }=\varepsilon_{\infty }-\frac{\omega_{p}^{2}}{\omega \left(\omega +{i\gamma }_{D}\right)}+\frac{f_{L}\omega_{L}^{2}}{\omega_{L}^{2}-\omega^{2}-i\gamma_{L}\omega }$$

where *ɛ*_∞_ is the background permittivity at high frequency, *ω*_*P*_ is the plasma angular frequency, *γ* is the carrier damping rate, *f*_*L*_ is the strength of Lorentz oscillator, and *ω*_*L*_ is the resonance frequency. In practical applications in ellipsometry, Drude, and single-peak Lorentz components are expressed as Equations (2) and (3):

(2) $$\varepsilon_{\text{Drude}}=P-\frac{{\left(E_{P}/K_{\text{zero}}\right)}^{2}\lambda^{2}}{1+{\left(\lambda \left(1/\tau \right)\right)}^{2}}+i\frac{\left(1/\tau \right){\left(E_{P}/K_{\text{zero}}\right)}^{2}\lambda^{3}}{1+{\left(\lambda \left(1/\tau \right)\right)}^{2}}$$

(3) $$\varepsilon_{\mathrm{Lorentz}}=\frac{A\lambda^{2}\left(\lambda^{2}-{\left(K_{\text{zero}}/E_{0}\right)}^{2}\right)}{{\left(\lambda^{2}-{\left(K_{\text{zero}}/E_{0}\right)}^{2}\right)}^{2}+\Gamma^{2}\lambda^{2}}+i\frac{A\lambda^{2}\Gamma }{{\left(\lambda^{2}-{\left(K_{\text{zero}}/E_{0}\right)}^{2}\right)}^{2}+\Gamma^{2}\lambda^{2}}$$

where P is the Drude background parameter, A is the intensity of the Lorentz oscillator, 1/τ is the mean free path, $E_{P}$ and $E_{0}$ are the energy of plasma wavelength and central wavelength of the resonance, respectively, Γ is the width of the peak, *K*_zero_ = 1.24 eV. By adjusting these parameters during the simulation, one can obtain the real and imaginary permittivities for Ag-ITO films.

The material parameters in the model can be expressed by Equations (4) and (5):

(4) $$\omega_{P}=\sqrt{ne^{2}/m^{*}\varepsilon_{0}}$$

(5) $$\gamma_{D}=1/\tau_{s}$$

where *n*, *e*, *m,* ^*^ and *ɛ*_0_ are carrier concentrations, electron charge, effective electron mass, and free space permittivity, respectively, and $\tau_{s}$ represents the scattering time. Considering the ENZ point where the real permittivity crosses zero at the screened plasma frequency $\varpi_{P}$, the relation between *ϖ*_*P*_ and *ω*_*P*_ is derived from Equation (6) [36]:

(6) $$\varpi_{P}=\omega \left(\varepsilon^{\prime }=0\right)=\sqrt{\omega_{P}^{2}/\varepsilon_{\infty }-\gamma_{D}^{2}}$$

More importantly, the requirements for supporting SPP propagation at the films/air interface is often considered as $\varepsilon^{\prime }<-\varepsilon_{\mathrm{air}}=-1$, and the corresponding frequency is so-called surface plasmon frequency which can be written as Equation (7) [36]:

(7) $$\omega_{\mathrm{SPP}}=\omega \left(\varepsilon^{\prime }=-1\right)=\sqrt{\omega_{P}^{2}/\left(\varepsilon_{\infty }+1\right)-\gamma_{D}^{2}}$$

When $\gamma_{D}\ll \omega$, the damping coefficient has limited effects on *ω*_*SPP*_ and can be dropped in Equation (7), so the allowed frequency of SPP is restricted in the range of $0<\omega <\omega_{P}/\sqrt{\varepsilon_{\infty }+1}$. However, if $\gamma_{D}$ is comparable to ω, especially at NIR wavelengths, the approximation is no longer valid.

### 2.4. Effective medium approximation

The effective medium theory is used to model the Ag-ITO composite films as an optically quasi-homogeneous material comprising of silver and ITO particles, with an effective permittivity *ɛ*_eff_ to represent the average optical response. Several models can be considered for modeling [37], and the relations between the permittivities can be jointly expressed as:

(8) $$\left(\varepsilon_{\text{eff}}-\varepsilon_{h}\right)/\left(\varepsilon_{\text{eff}}+2\varepsilon_{h}\right)=\sum_{i}f_{i}\left(\varepsilon_{i}-\varepsilon_{h}\right)/\left(\varepsilon_{i}+2\varepsilon_{h}\right)$$

Here, *ɛ*_*i*_ and *ɛ*_*h*_ are the permittivity of the constituent and host, *f*_*i*_ is the volume fraction of the *i*th constituent, which was calculated based on atomic ratio (obtained by fitting the element peaks in XPS spectra) and the ratio of atomic (or molecular) weight to density of two elements. When silver (*ɛ*_*i*_ = *ɛ*_*Ag*_) particles are treated as small spheres diffusing inside the host continuum ITO (*ɛ*_*h*_ = *ɛ*_ITO_), Equation (8) becomes Maxwell-Garnett model (referred to as MG model) and it is only impactful when the radius of silver particles is much smaller than the incident wavelength. There is another case when the constituents/host are chosen as silver and ITO (*ɛ*_*i*1_ = *ɛ*_Ag_,$\varepsilon_{i2}=\varepsilon_{\text{ITO}}$)/air (*ɛ*_*h*_ = 1), respectively, Equation (8) becomes extended Maxwell-Garnett model (referred to as EMG model). It assumes that Ag and ITO particles are mixed at their respective filling factors in the void [27,37]. Therefore, EMG model is chosen for further investigations in the following effective medium approximation session.

## 3. Results and discussions

### 3.1. Microstructure analysis of the growth

We obtained the ITO-based composite films with different silver contents. In order to ascertain the high-transmittance and low-loss properties of composite films, the silver amount are set to be small (under 3 at.%) on purpose. The specific silver atomic ratios are: 0.15, 0.44, 0.95, 1.37, 2.17, and 2.74 at.%. To represent the changing process in microstructure, films with 0.15, 0.95, and 2.74 at.% Ag were selected for analysis.

**Figure 1.**
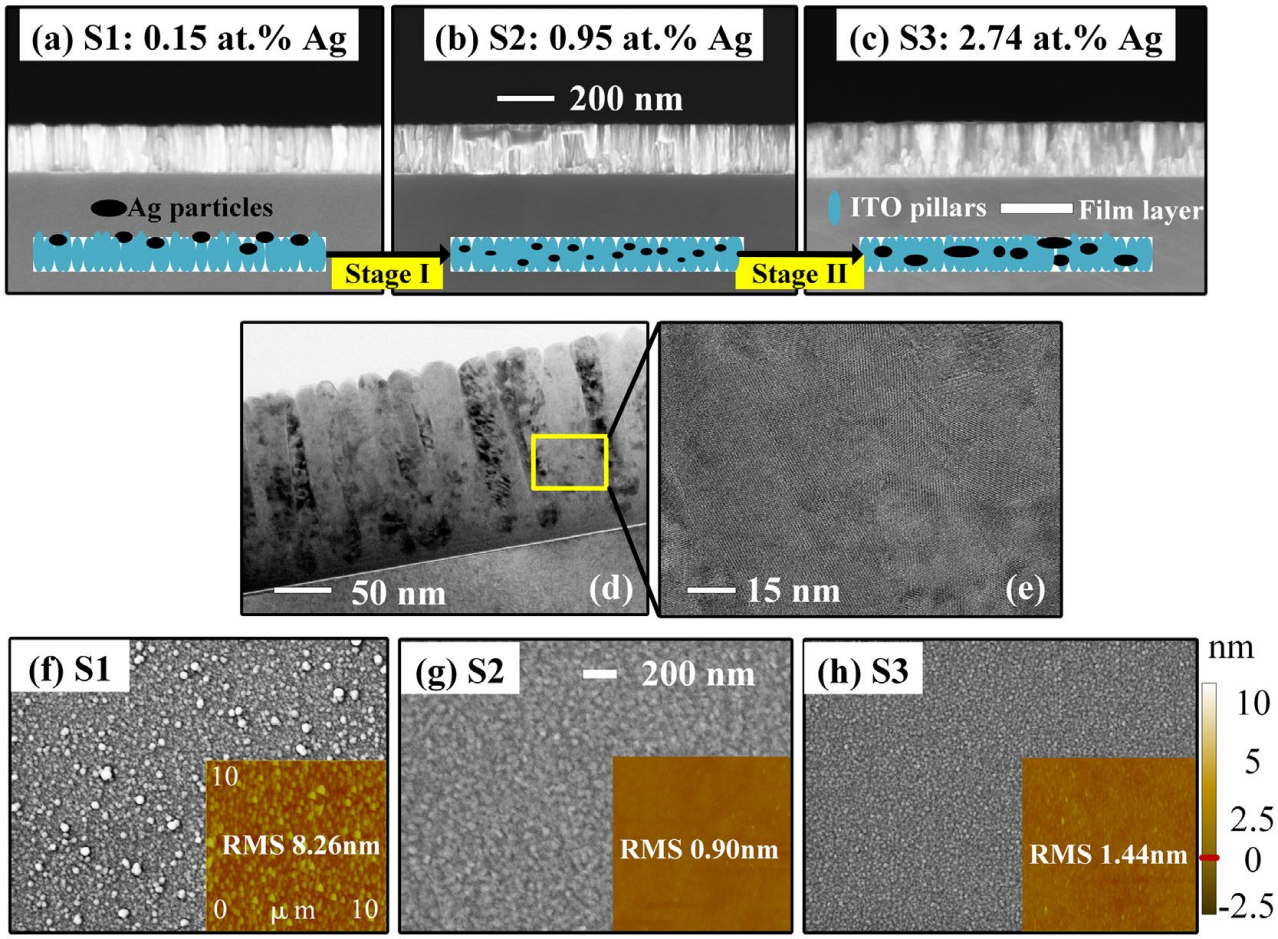
Surface and cross-sectional morphologies of as-deposited Ag-ITO films with different silver contents, obtained from (a)–(c): Cross-sectional SEM (insets: schematic of the distributions for Ag and ITO particles and different growth stages); (f)–(h): Surface SEM (insets: AFM images) and (d)–(e): TEM.

Notes: Films with 0.15, 0.95, and 2.74 at.% Ag were selected for analysis and labeled as sample 1(S1), sample 2(S2), and sample 3(S3), respectively (the same below). Parts of (a)–(c) and (f)–(h) are reproduced from [55].

Figure 1 demonstrates the effect of varying silver contents on the microstructure for as-deposited films. In SEM cross-sectional views (Figure 1(a)–(c)), all the films exhibit good thickness uniformity, and nano-column structures of ITO films are clearly observed. No significant differences have been observed in TEM results for different samples. Figure 1(d)–(e) show representative TEM images, and their detailed structure in the enlarged views. Pillar-like nanostructure vertical to the surface of the substrate indicates strong preferred orientation of the films in (d), while polycrystalline lattices with different orientations are observed in (e). Elements of ITO are found well-distributed, while silver is too few for detection owing to its small amount and weak crystallinity, which will be revealed in the XRD results since no ordered phase for Ag is detected.

The corresponding SEM surface views are shown in Figure 1(f)–(h). For the sample with less than 0.2 at.% Ag (labeled as sample 1), the silver grains are found spreading on the upper layer or on the surface (bright bulks determined from energy dispersive spectrometer). The grain size is comparatively large and the overall distribution is thus in disorder (Figure 1(f)). This is mainly caused by our fabrication technique when the sputtering power for silver is too small (RF 5 W) compared with ITO (DC 300 W). The sputtered silver particles are thus inhomogeneously distributed and phase-separated from ITO particles. Nevertheless, silver and ITO particles become more densely packed when the atomic ratio of silver increases to nearly 1 at.% (labeled as sample 2). Silver particles with comparatively small sizes are distributed within the ITO layer, resulting in so-called Ag-ITO composite films (Figure 1(g)). After that, the additional silver incorporates into the ITO layer (labeled as sample 3) and starts to aggregate (Figure 1(h)). The insets of Figure 1(a)–(c) show schematics of the growth process. Black and blue ellipses stand for Ag and ITO. Pillar-like ITO structures constitute the majority of the composite layer, while tiny amount of silver exists in the form of dispersed particles, tuning the microstructures of the composite layer. Hence, from the three typical microstructures of Ag-ITO films obtained above, there exists the most appropriate silver content within the certain limit for the ideal composite status. (1 at.% for our series of films).

The reasons for the changes in microstructures can be attributed to the following principles [37]: The procedure between sample 1 and 2 is the initial stage I. For co-sputtering, it represents the transition from inhomogeneous status to the formation of composite films. In correspondence, there are two types of models describing this transition: the first is the aggregate structure, in which tiny amount of silver and dominating ITO are segregated in isolated structures. ITO forms the percolation paths and undertakes the work to conduct the carriers offered by Ag and ITO; the second is the separated-grain structure, in which silver with smaller sizes disperse among the continuous ITO layer. At stage II as the silver ratio increases (represented by the procedure between sample 2 and 3), more and more silver induces the agglomeration and enlargement of grain sizes. The role of silver in the composite film is rather interesting, since further electrical and optical measurements confirm that larger-grain silver may acts as scattering centers and even traps for carriers, thus deteriorating the electrical properties of composite films and red-shifting the ENZ region.

The insets of Figure 1(f)–(h) show the evolution of 2D AFM micrographs. The results illustrate the evolvement of the RMS roughness for sample 1–3. The optimal smooth film (sample 2) possesses the lowest roughness of 0.9 nm. Either reducing or introducing silver content lead to coarse films and higher RMS value. This echoes with the surface SEM images and the two-stage growth mechanism.

Here we underline the nature for this ‘optimization’ phenomenon as that the optimal film composition for structural homogeneity is existed objectively, though not determinant (e.g. may be another amount of silver content for different fabrication apparatus). We mainly focus on the tuning ENZ region and plasmonic properties by optimizing the silver content and post-annealing parameters.

**Figure 2.**
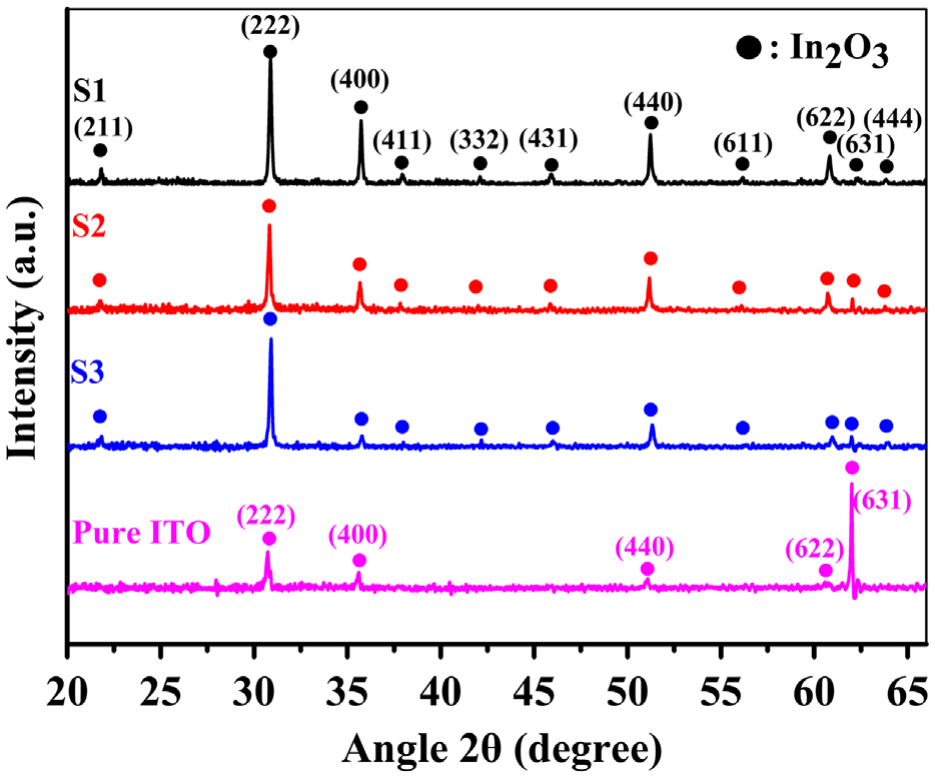
XRD patterns of Ag-ITO films with different silver contents compared with pure ITO films under identical fabrication conditions (see labels for details).

Notes: The patterns for sample 1–3 and ITO are depicted in black, red, blue, and purple curves, respectively. The peak positions of In_2_O_3_ are represented as circles (●).

To evaluate the silver-induced structural changes, we analyzed the X-ray diffraction (XRD) results. As shown in Figure 2, it can be inferred that the polycrystalline microstructure of Ag-ITO films is highly related to the silver introduction and atomic ratio. For pure ITO films, the diffraction peaks at 30.5°, 35.4°, 51.0°, 60.7°, and 62.2° correspond to the (2 2 2), (4 0 0), (4 4 0), (6 2 2), and (6 3 1) planes of the polycrystalline In_2_O_3_, respectively (JCPDS#06–0416), indicating that Sn^4+^ replaced the In^3+^ site or incorporate in the interstitial positions of In_2_O_3_ [22]. The peak of (631) plane is rather strong in the ITO-only layer, which differs from the majority of the previous reports [24,38,39] with higher (222) peak. For sample 1–3, more diffraction peaks representing crystalline In_2_O_3_ emerge, for instance, (211) at 21.5°, (411) at 37.7°, (332) at 41.8°, (431) at 45.7°, (611) at 55.9°, (444) at 63.7°, and etc. (JCPDS#06–0416). Besides, the Ag-ITO samples are not exhibiting peaks with high signal-over-noise value corresponding to Ag (JCPDS#04–0783). We may predict that silver is not in an ordered phase. However, it is noteworthy that the (222), (400), (440), and (622) peaks for sample 1–3 are enhanced obviously compared to pure ITO films. With the increase of silver ratio, the (400), (440), and (622) peaks weakens monotonically and the (222) peak declines for sample 2 and then inclines afterward. We calculated the intensity ratio (222)/(400) and the result shows a gradual ascent: 1.959, 2.854, and 7.497 for sample 1, 2, and 3, respectively.

From the above outcomes we can conclude that the incorporation of silver not only affects the macroscopic structure of ITO, but also induces the enhancement of diffraction peaks for several preferred orientations of In_2_O_3_. When the amount of silver is kept under a certain limit (in this case <3 at.%), silver content improves the dominating role of (222) peak and suppresses the other polycrystalline structures relatively.

### 3.2. Permittivity (*λ*_*c*_) and ENZ region tuning

**Figure 3.**
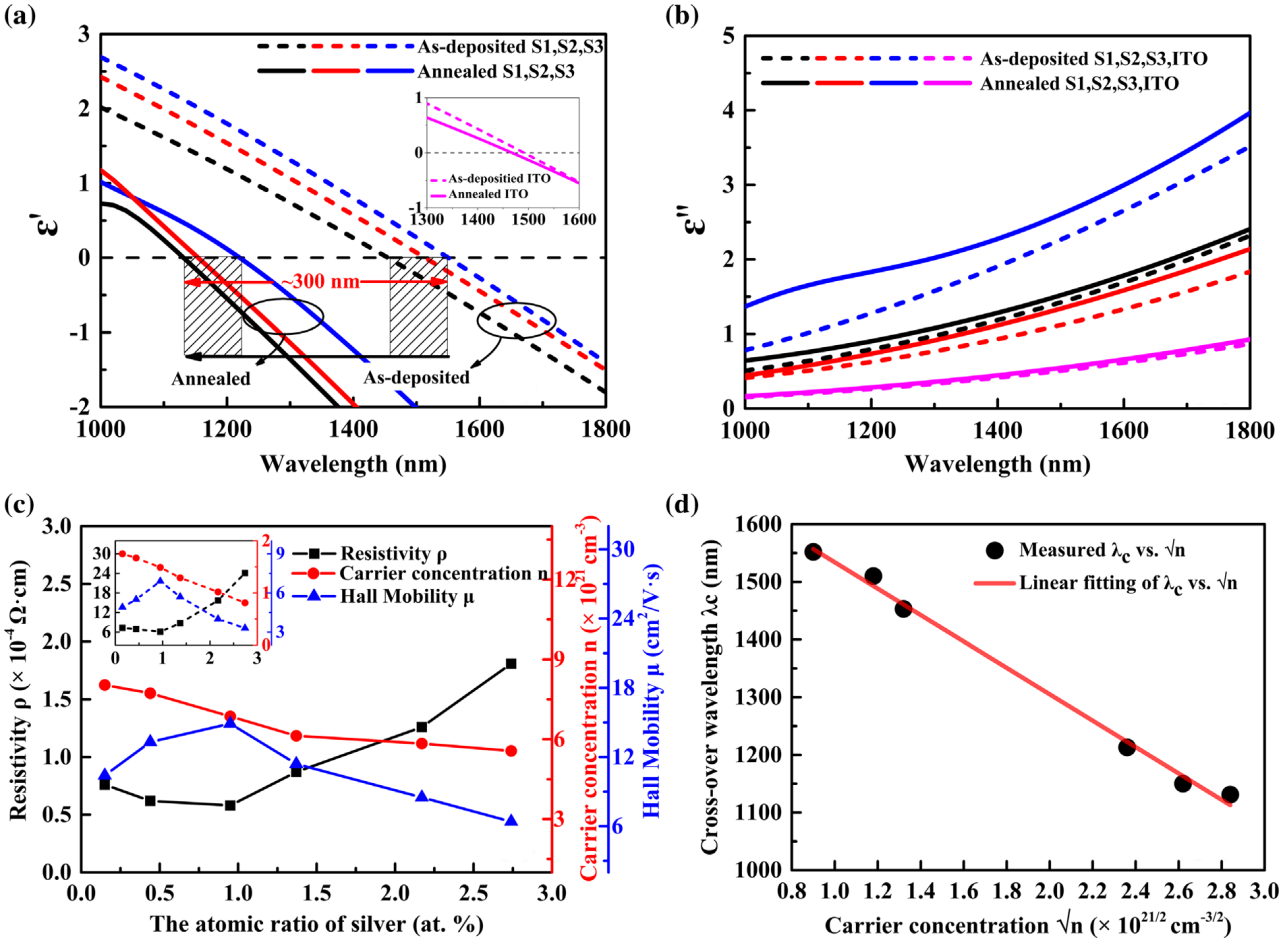
Wide range of *λ*_*c*_ and ENZ tuning can be achieved by optimizing the silver content and post-annealing parameters: (a) Real permittivity for Ag-ITO films (inset: real permittivity for pure ITO films); (b) Imaginary permittivity; (c) Hall measurements for the annealed films (inset: as-deposited films); (d) Experimental data and linear fitting of *λ*_*c*_ vs. square root of carrier concentration.

Notes: Parts of (a) and (b) are reproduced with permission from [55].

The optical properties, and plasmonic properties in particular, can be manipulated utilizing many experimental methods, such as changing deposition conditions and varying the stoichiometry of the composite films [14,21,38,40–43]. One of the valuable assets of Ag-ITO composite films is that the plasmonic properties and ENZ regions can be tuned readily by altering atomic content of silver and rapid thermal annealing (RTA). The results of dielectric permittivity extracted from spectroscopic ellipsometry using a Drude–Lorentz model are summarized in Figure 3. The dashed lines represent the as-deposited films, while the solid lines refer to the annealed samples. Pure ITO films deposited under same conditions are reproduced as reference.

As can be seen from the comparisons between as-deposited films in Figure 3(a), the inclusion of Ag shifts the real permittivity curves. Figure 3(b) depicts the imaginary permittivity, among which increases a relative reasonable amount with the increase of silver content and after RTA. We found that the cross-over wavelength *λ*_*c*_ vary from 1453 nm (sample 1) to 1552 nm (sample 3), with only 2.6 at.% alternation of Ag content. So detailed adjustment of *λ*_*c*_ within 100 nm can be achieved through this method. However, to maximize the advantages of silver-incorporation in the area of ENZ manipulation, wider range of tunability covering as much NIR wavelengths as possible is desirable. Substantial parameters are dedicated to accomplish this goal [43,44]. Here, we use rapid post-annealing. The solid curves in Figure 3(a) show that *λ*_*c*_ shifts to shorter wavelengths for more than 300 nm after RTA (Sample 1: From 1453 to 1131 nm; sample 2: From 1512 to 1152 nm; and sample 3: From 1547 to 1218 nm). The Ag-ITO films all display remarkable tunability even if RTA lasts for only one minute. So RTA can be treated as the main tuning factor for all kinds of ITO-based composite films. Wider range of RTA conditions (e.g. longer time, higher temperature, or annealing speed) also help in tuning the permittivity to meet unique requirements. But enlarging silver particles after stronger thermal treatments may result in uncontrollable status which is difficult for modeling. Hence, 1 min and 400 °C were chosen as the specific parameters. Oppositely, as can be seen from the inset of Figure 3(a), pure ITO films after rapid annealing are far less tunable than Ag-ITO films and the ENZ region is almost unchanged.

Notably, the counter-intuitive part lies in the effect of silver. Judging from the varying trend in Figure 3(a), augmenting silver content will cause consecutive red-shifting of *λ*_*c*_. Since *λ*_*c*_ is in reverse proportion to the square root of carrier concentration, i.e. $\lambda_{c}\propto 1/\sqrt{n}$ [5] which means that carrier concentration reduces instead of increasing after adding more silver. We intend to explain this phenomenon in experimental and theoretical aspects.

In experiments, Figure 3(c) shows the electrical properties obtained from Hall measurement which confirms this results. With the increase of silver content, carrier concentration *n* drops monotonically for both as-deposited (inset) and annealed films. There is a significant rise in *n* after annealing due to the affection by silver. The more significant result is that there exists an optimized silver content corresponding to the lowest resistivity and largest hall mobility, and as for our experiments, nearly 1 at.%. It correlates with the structural analysis in which 1 at.% silver is confirmed to be at the end of stage I when silver particles diffuse into the ITO layer, thus so-called composite film is formed. The peaks for Hall mobility (blue curves in Figure 3(c)) also appear at the optimized condition, giving rise to the high conductivity though the carrier concentration decreases. Figure 3(d) selects the typical points of *λ*_*c*_ vs. $\sqrt{n}$ for S1, S2, and S3, and then fits the data linearly. The goodness-of-fit portrayed by R^2^ is larger than 0.993, indicating that *λ*_*c*_ and $\sqrt{n}$ satisfy the reverse relations.

In theory part, two stages mentioned in structural analysis possibly accounts for this phenomenon. Even though silver seems to bring in free electrons, the carrier concentration reaches the peak at the initial status of stage I, when ITO dominates the percolation paths for carrier mobility and bulky-grain silver separated from ITO particles also acts as conductor supplier, thus elevating the carrier concentrations. With the increase of silver amount at stage II, silver grains are gradually mixed in the ITO continuum. Ag^+^ may serves as the same role of Sn^4+^ which affects the spatial arrangements and structures of ITO-only layers. There are more interfaces between larger silver particles and ITO particles which scatter and block out carriers, thus reducing the effective carrier concentrations. The crystalline status of continuous ITO and mobile carriers is then strongly impacted by random dispersion of innumerable silver particles. The lowering of diffraction peaks for other orientations of In_2_O_3_ except (222) may also results from such silver affection (Figure 2).

The wide range tunability after RTA is due to the changing status of the embedded silver. The thermal treatment in a short time not only improves the crystallinity of ITO, but more importantly, makes the scattered silver particles start to form the percolation path and thus increase the carrier amount accounting for delocalized movements. Such predictions are reflected in the abrupt rise of carrier concentration in Figure 3(c).

Additionally, the transmittance and absorption spectra over visible-NIR range (400–2000 nm) of the composite films and ITO films are shown in Figure S2 of the supplementary materials. We can observe that for as-deposited films, silver-introduction results in decrease of overall transmittance, especially in the visible region. This is due to the absorption induced by silver nanoparticles as can be judged from the absorption spectra. Markedly, the NIR transmittance of sample S1 and sample S2, which have silver content lower than 1 at.%, are comparable with that of ITO. The structural optimized sample S2 has smaller absorption than S1 (with fewer silver) at wavelengths over 1300 nm, which supports the two-stage structural analysis and the explanations of Ag’s role. Annealing coarsen the Ag and ITO particles, creating more scattering centers and boundaries. Meanwhile, larger silver particles dispersed in ITO layer result in the significantly reduced transmission and increased absorption before 600 nm. The overall visible transmittance for annealed S1, S2, and S3 samples is approximately 76, 68, and 62%, respectively, while for as-deposited films, it is slightly higher, at 79, 74, and 65%, respectively.

The above results demonstrate a plasmonic material that can be controlled through composition (silver content) and rapid post-annealing with extreme tunability of ENZ region and reasonable loss for applications.

### 3.3. Effective medium approximation for ENZ tuning simulation

**Figure 4.**
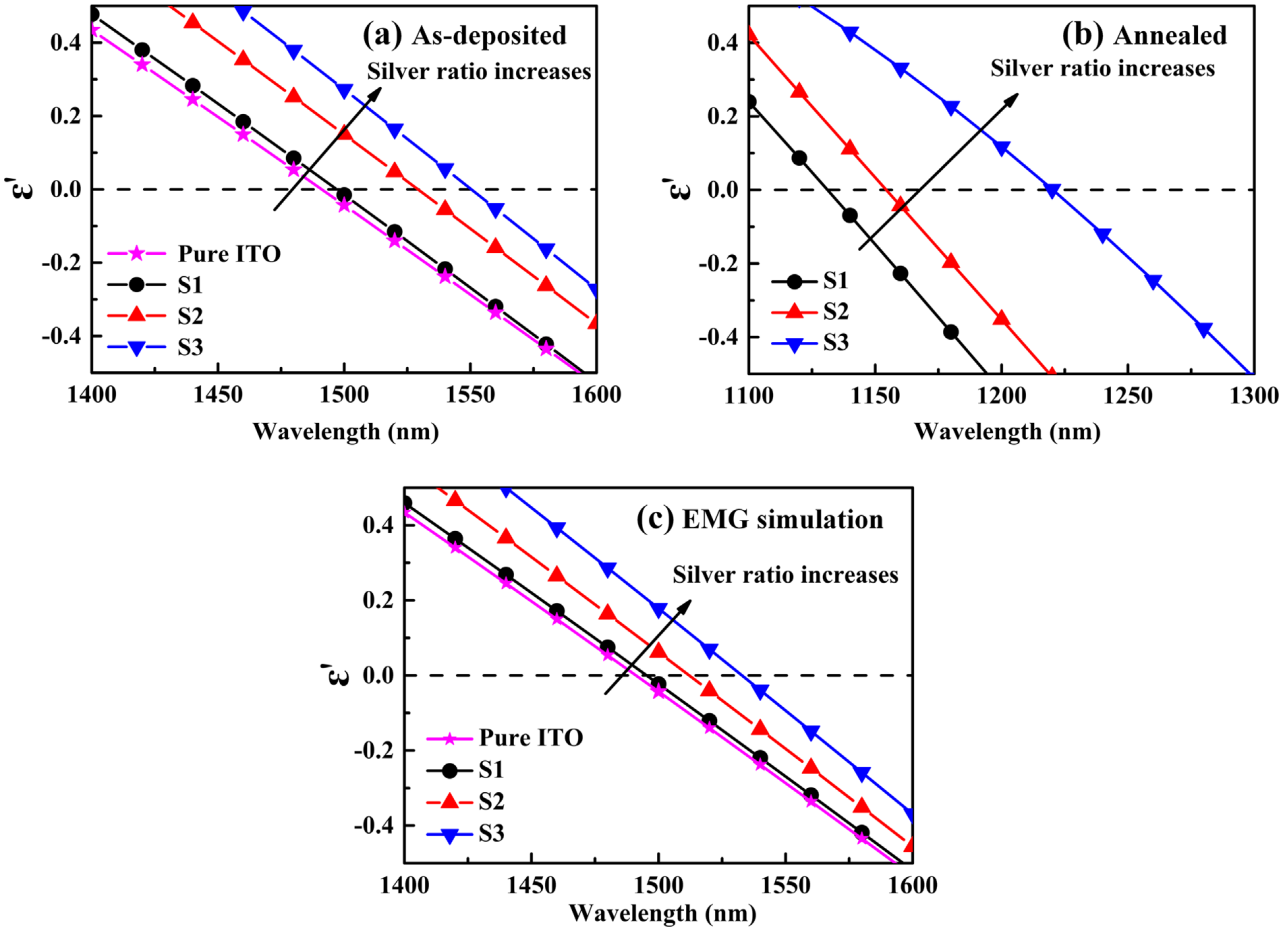
ENZ region for Ag-ITO films with different silver contents: (a) Experimental data of as-deposited Ag-ITO films; (b) Experimental data of annealed Ag-ITO films; (c) Effective medium approximation based on extended Maxwell-Garnett model which takes silver and ITO as constituents and air as host.

Notes: Data for pure ITO films without Ag are also plotted in (a) and (c) for comparisons. In (b) the pure ITO curves are outside the wavelength range and not displayed.

To figure out the mechanisms for the counter-intuitive ENZ-shifting, the topical ENZ region for Ag-ITO and pure ITO films are shown in Figure 4(a) and (b). Effective medium approximation (EMA) was used to simulate such ENZ-shifting behavior. Figure 4(c) shows the effective permittivity of Ag-ITO films by applying extended Maxwell-Garnett (EMG) model and treating the silver and ITO particles as constituents embedded in air. The results reveal a long-wavelength shift which coincides with the results of Figure 4(a) and (b). The wavelength range predicted by simulation coincides with that of as-deposited films, but not annealed films. That is due to the coarser and larger silver particles after annealing, which starts to affect and even dominate the optical properties. In the annealed case, another model should be chosen for interpreting the silver-based behavior.

Though the EMG model is well suited for determining the shifting trend as long as the continually increasing silver content is above the inhomogeneous amount and no annealing is conducted, there’s some discrepancy between experimental data and EMA results. The major mismatch is that the shifting behavior cannot be described quantitatively. It is without doubt that the practical fabricated films cannot be totally approximated by the EMA method, since the conditions for EMG model is hard to be fulfilled, which poses limitations in validity [37]. This limitation will only be eliminated if much smaller silver spheres are distributed randomly among the ITO continuum and homogeneous status at an atomic level is assured. Moreover, the parameters used in the simulation, especially the volume ratio and index of silver particles are adopted from theoretical values rather than measured experimentally, which also introduce extra deviations. Although the violation of the size limit and inaccurate simulation results necessarily happens for the composite films, it doesn’t mean the breakdown of extended Maxwell-Garnett theory in the determination of permittivity for Ag-ITO films. It is worth stressing that the ENZ-shifting trend of Ag-ITO films can be well-defined by EMG model.

### 3.4. Prism-coupled SPP verifications

The experimental investigations of the near-infrared SPP excitations on Ag-ITO films were performed to verify the applicability for plasmonic devices, especially that operating at telecommunications wavelength. Considering the operating requirements, the cross-over wavelength *λ*_*c*_ needs to be matched with the SPP excitation conditions, in which SPPs are described by their dispersion relations [9]. The real component determines the SPP wavelength and can be written in Equation (9):

(9) $$k_{\mathrm{SPP}}^{\prime }\left(\omega \right)=k_{0}\sqrt{\varepsilon_{d}^{\prime }\left(\omega \right)\varepsilon_{m}^{\prime }\left(\omega \right)/\left(\varepsilon_{d}^{\prime }\left(\omega \right)+\varepsilon_{m}^{\prime }\left(\omega \right)\right)}$$

And SPP wavelength is expressed as:

(10) $$\lambda_{\mathrm{SPP}}\left(\omega \right)=2\pi /k_{\mathrm{SPP}}^{\prime }=\lambda_{0}\sqrt{\left(\varepsilon_{d}^{\prime }+\varepsilon_{m}^{\prime }\right)/\varepsilon_{d}^{\prime }\varepsilon_{m}^{\prime }}$$

In the above equations, $k_{0}=2\pi /\lambda_{0}$ denotes the free-space light wavevector, $\varepsilon_{d}^{\prime }$, and $\varepsilon_{m}^{\prime }$ are the real permittivities of the air and film, respectively. It is evident that the permittivity for films under the wavelength of incident light has a threshold value ($<-\varepsilon_{d}^{\prime }$). So the cross-over wavelengths need to be shifted to shorter values by post-annealing.

**Figure 5.**
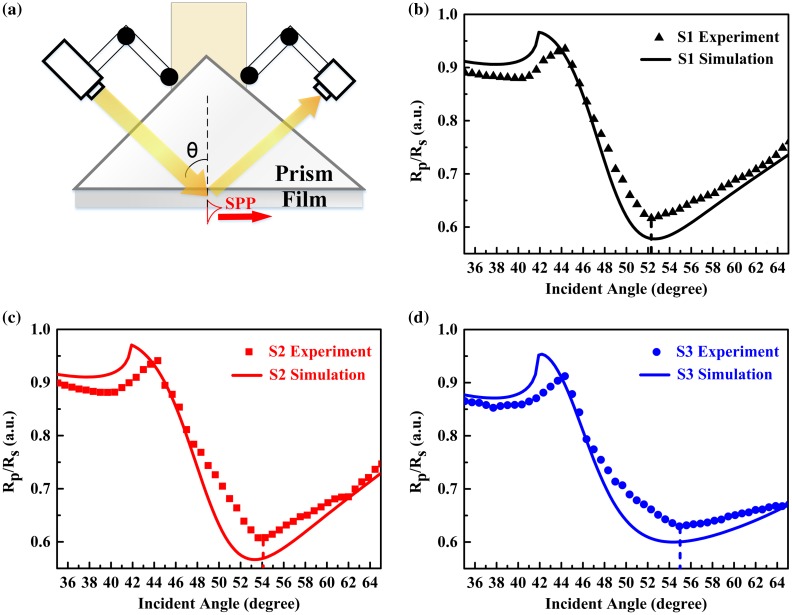
Reflectance curves for annealed Ag-ITO films vs. incident angle at the wavelength of 1550 nm: (a) Schematic of the prism-coupling configuration; (b)–(d) Experimental results by prism-coupling; and simulation by transfer-matrix method for sample 1, 2, and 3, respectively.

A Kretschmann-Raether configuration was implemented for prism coupling (see Figure 5(a)). Polarized, collimated fiber laser beam at 1550 nm (AFC Inc. BBS-1550, Richardson, TX, USA) was applied to illuminate the annealed Ag-ITO films through a 45° K9 glass prism. Then, the reflected light was collected and measured with an InGaAs detector (Newport Model 818-IS-1, Irvine, CA, USA) connected to a power meter (Newport Model 1830-C, Irvine, CA, USA). The angular scanning is achieved in the self-assembly angle-variant system (SPL RTM-R1, Hangzhou, China) by means of rotating incident beam as well as detector with respect to the fixed sample attached to the prism mounted on the platform. The reflected intensity of the composite films is recorded between 35° and 65° with a step of 1°.

The results are summarized as the dotted lines in Figure 5(b)–(d), which show reflectance curves for Ag-ITO films with various silver ratios. *R*_*p*_ and *R*_*s*_, the reflectance spectra of TM-polarized and TE-polarized light were obtained respectively and are calculated as the ratio of *R*_*p*_/*R*_*s*_. We use this ratio for further analysis since *R*_*s*_ is without characteristics at this spectra [45]. Previously, the observation of broad SPP dips in pure TCO films has been reported [36,40,45,46]. Those discussions illustrated wavelength-of-incidence-, material-, and thickness-dependent SPP resonances and as a supplement, our study clearly shows the SPP existence on Ag-ITO composite films as a function of silver ratio. For each curve, the total internal reflection appears at about 44°. The reflectance dips, from 52° to 56° when increasing silver ratio from 0.15 to 2.74 at.%, reveal pronounced SPP resonances. Besides, the depth of the dips is related to the imaginary permittivity *ε*″ of the films, i.e. smaller *ε*″ means less dissipative loss, which indicates that more energy can be sustained to excite SPP and larger dip will be observed. For SPR motivation, the ideal material is lossless metallic component. The reflectance curve will then be a narrow and sharp dip at the resonance wavelength. Large *ε*″ (large loss) will not lead to lower dip reflectance, but weakens the SPR effect which will be directly seen from the shallowing depth and broadening width of the reflectance dip. So, the dips for samples with less silver content are deeper.

To validate the experimental curves, numerical simulations [47] based on transfer-matrix method were performed by fitting the data retrieved from sample measurements (see the solid curves in Figure 5(b)–(d)). Here, we estimated the thickness of each film to be 200 nm. The dielectric constants at 1550 nm were calculated to be *ε*_1_ = –3.51 + 1.55i, *ε*_2_ = –3.23 + 1.46i, and *ε*_3_ = –2.42 + 2.45i, respectively. There are some mismatches (e.g. total reflection edge positions, depth of valleys, and trends in certain region) due to fabrication and measurement imperfections. For example, the sensitivity of the detector is not enough for small measuring resolution and manual recording in the SPP-dip range, in which *R*_*p*_ and *R*_*s*_ are both small and the ratio of *R*_*p*_–*R*_*s*_ is thus inaccurate. However, the dip positions of the simulation results, which are of significance to the SPP properties, are in agreements with experimental outcomes. This also confirmed that the Drude–Lorentz model and parameters of retrieving permittivities were reasonable for Ag-ITO films.

## 4. Conclusions

In conclusion, suitable alternatives to metal and pure TCO films as NIR plasmonic materials – Ag-ITO co-sputtered composite films, were synthesized by tailoring the silver atomic ratio ranging from 0.15 to 2.74%. The mechanisms for the transformation of films structures, crystallinity, and preferred orientations are elaborated. A more important issue is that the Ag-ITO films exhibit exceptional optical properties, especially that their cross-over wavelengths *λ*_*c*_ (i.e. epsilon-near-zero region) are tunable by altering the silver content or by applying rapid post-annealing. Recently, giant optical nonlinearity at ENZ region has been observed in pure TCO (ITO and AZO) films and their nanostructures (nanorods) [13,14,48,49]. There is also high potentiality to exploit our composite films or structures for enhancing ENZ-nonlinear effects, which will induce large and ultrafast refractive index change. Thus, such materials with optical-induced nonlinear response are applicable to all-optical information processing, plasmonics, and nonlinear optics. Additionally, the niche applications for the composite films are the components of light absorbers in the visible and NIR region. Reports about ENZ TCO thin films as perfect absorbers have been proposed recently [50–54]. Due to the larger loss induced by silver inclusion, the ENZ composite films can be engineered and combined with metals to be applied in absorbers. Effective medium approximation based on extended Maxwell-Garnett model is applied to describe the tuning trend and the red-shifting of *λ*_*c*_ (vs. the increase of silver content) is definitely matched with the prediction of the model. Moreover, Ag-ITO films were demonstrated to support SPP resonances by prism coupling at optical communication wavelength. Such variability of the plasmonic properties could be used to enable new controllable NIR devices.

## Disclosure statement

No potential conflict of interest was reported by the authors.

## Funding

This work was supported by the National Natural Science Foundation of China [grant number 61575176]; the National Basic Research Program of China (973 Program) [grant number 2013CB632104]; the Research Foundation of State Key Laboratory of Modern Optical Instrumentation [grant number MOI201701].

## Supplemental data

Supplemental data for this article can be accessed here. [https://doi.org/10.1080/14686996.2018.1432230].

## Supplementary Material

Supplementary_materials.docx
