# Supplementary material for: Tunable near-infrared epsilon-near-zero and plasmonic properties of Ag-ITO co-sputtered composite films
Source: Sci Technol Adv Mater. 2018 Feb 19;19(1):174–84. doi: 10.1080/14686996.2018.1432230 (PMC5827799; doi:10.1080/14686996.2018.1432230)
Supplement: Supplementary_materials.docx [file TSTA_A_1432230_SM5315.docx]

**Supplementary materials**

Supplementary Note:

To fully evaluate the potential of Ag-ITO composite films for NIR plasmonic applications, the SPP profile, which has been widely applied as a gauge for comparing various plasmonic materials, are calculated based on the parameters derived from Figures 3(a) and (b). The comprehensive method to measure all surface plasmon waves is to consider the balance between propagation lengths (i.e. the distance over which the SPP mode propagate until the intensity falls down to $1/e$ of the initial magnitude) and degree of confinement (i.e. penetration depths into materials) [19].

As Figures S1 (a) and (b) shows, the propagation length ($\delta_{Prop}$) is defined as [9]:

$\delta_{Prop}=\lambda_{0}\frac{\left( \varepsilon_{m}^{'} \right)^{2}}{2\pi\varepsilon_{m}^{''}}\left( \frac{1+\varepsilon_{m}^{'}}{\varepsilon_{m}^{'}} \right)^{3/2}$ (1)

where$\varepsilon_{m}^{'}$ and$\varepsilon_{m}^{''}$ represent the real and imaginary permittivities of the composite films, $\lambda_{0}$ stands for free space wavelength. It is evident from Figure S1(a) that the propagation lengths of SPP on the composite films after annealing at NIR wavelengths are significantly larger than that of ITO. At telecommunication wavelength, the SPP can propagate up to over 1 microns for the composite films. However, in Figure S1(b), the as-deposited films has limited SPP-propagation ability which is even worse than ITO. It is due to large loss induced by silver inclusion which leads to a large denominator $\varepsilon_{m}^{''}$ in equation (1), and the numerator (dominated by$\varepsilon_{m}^{'}$) is not large enough to counteract the influence. After annealing, $\varepsilon_{m}^{'}$ significantly blue-shifts with larger magnitude (more negative), thus result in increased propagation length.

While in Figures S1(c) and (d), the penetration lengths into the composite films are defined as [9]:

$\delta_{Conf}=\frac{\lambda_{0}}{2\pi}\left| \frac{1+\varepsilon_{m}^{'}}{\left( \varepsilon_{m}^{'} \right)^{2}} \right|^{1/2}$ (2)

This parameter determines the confinement of the surface wave at the supporting surface. As can be seen from the Figure S1(c), the penetration depth inside the composite film is slightly shorter than that for pure ITO film. The penetration depth diverges for the wavelength region in which the films is transparent to the incident light. When the incident wavelength is longer than$\lambda_{SPP}$, this confinement parameters are asymptotically approaching stable values. At longer wavelengths (approaching 2 μm), ITO can confine SPP at ~140 nm scale, which is 10 % larger than that of Ag-ITO films (100~125 nm). Both of the above results validate that annealed Ag-ITO composite films can confine and propagate SPP at NIR wavelengths better than ITO.


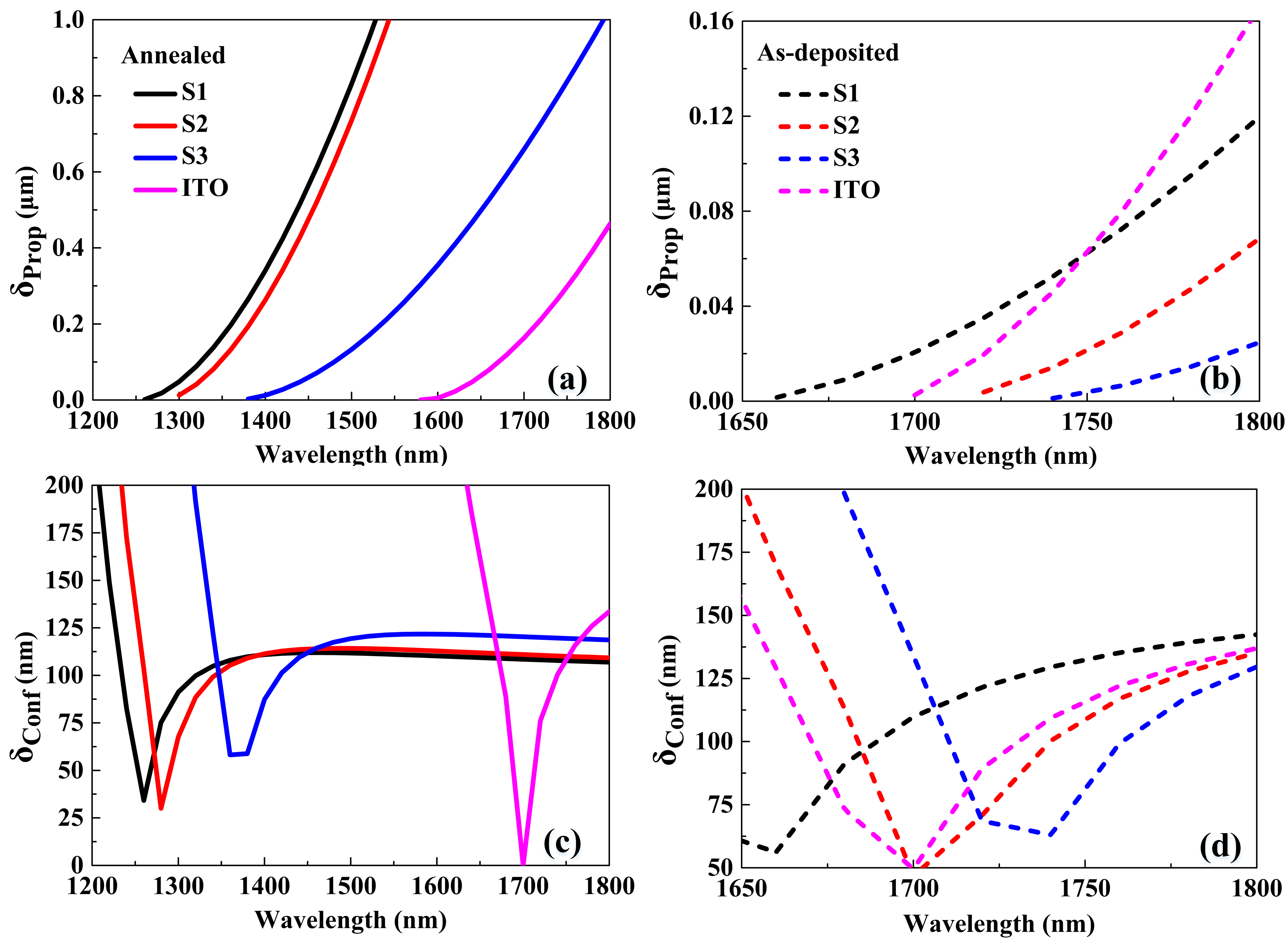


Figure S1. Two-dimensional criteria of plasmonics (SPP) for Ag-ITO composite films and pure ITO films: Propagation length$\delta_{Prop}$ for annealed films (a) and as-deposited films (b); Degree of confinement$\delta_{Conf}$ for annealed films (c) and as-deposited films (d).


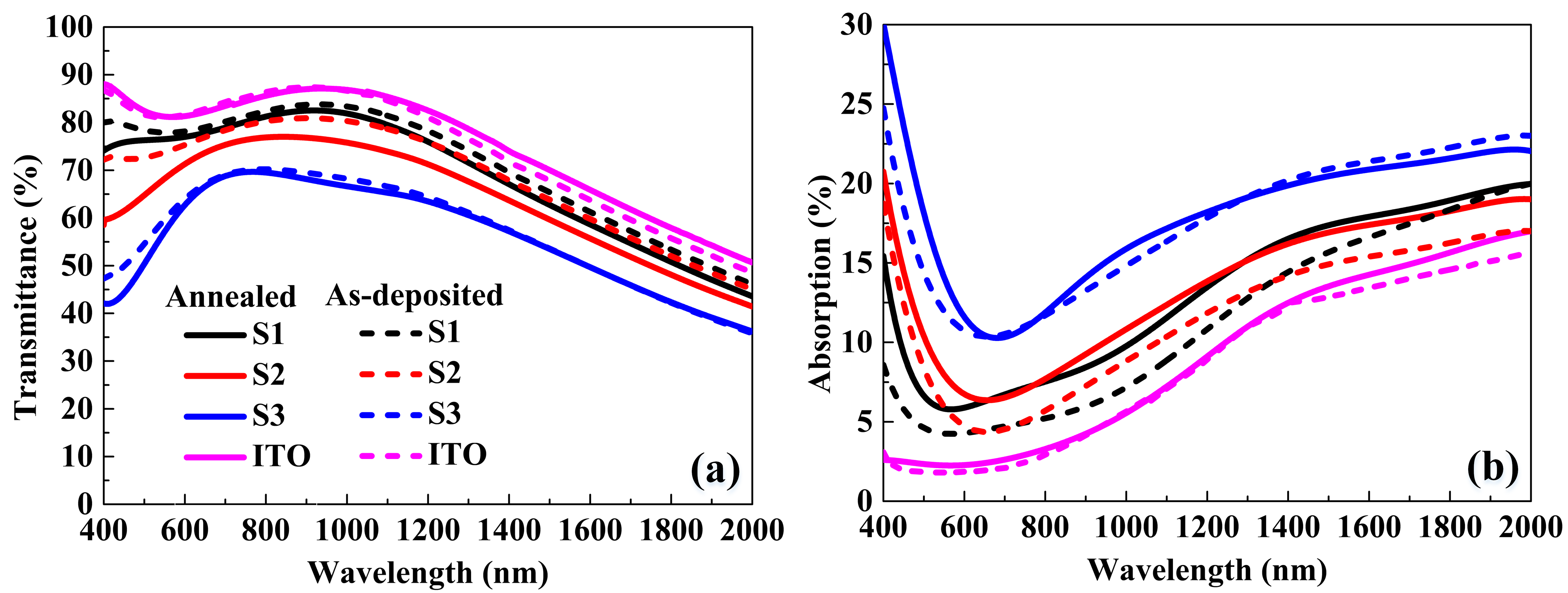


Figure S2. (a) Transmittance and (b) Absorption spectra (400~2000 nm) for Ag-ITO composite films and pure ITO films.
